# An Investigative Study of Hepatic Arterial Anomalies in a West Indian Population

**DOI:** 10.1155/2021/9201162

**Published:** 2021-10-15

**Authors:** Shamir O. Cawich, Alexander Sinanan, Maria Gosein, Neil Pearce, Rahul Deshpande, Fawwaz Mohammed, Vijay Naraynsingh, Maurice Fortune, Fidel Rampersad

**Affiliations:** ^1^Port of Spain General Hospital, Port of Spain, Trinidad and Tobago; ^2^Southampton University Hospital NHS Trust, Southampton, UK; ^3^Manchester Royal Infirmary, Oxford Road, Manchester M13 9WL, UK; ^4^Eric Williams Medical Sciences Complex, San Juan, Trinidad and Tobago

## Abstract

**Purpose:**

There are many known variations in the arterial supply to the liver. We sought to document the incidence and details of anomalies of the extrahepatic arteries in an unselected population in the West Indies.

**Methods:**

This study spanned 24 months. All 205 CT scans were evaluated at a hepatobiliary referral center in Trinidad and Tobago. We described the anomalies of the arterial supply to the liver using the conventional classification proposed by Michels.

**Results:**

205 CT scans were evaluated, and 112 persons (54.6%) had conventional Type 1 anatomy. However, compared to the incidence in the existing medical literature, we encountered a greater incidence of replaced right hepatic arteries (18.1% vs 11%; *P* 0.04) and a lower incidence of accessory right hepatic arteries (2.4% vs 7%; *P* 0.030).

**Conclusion:**

Although 54.6% of persons in this West Indian population have conventional hepatic arterial supply, the distribution of anatomic variants of the right hepatic artery is quite different to that seen in North American and European centers. We found a higher incidence of replaced right hepatic arteries and a lower incidence of accessory right hepatic arteries.

## 1. Introduction

The number of invasive surgical and endovascular procedures performed on the liver have increased over the past three decades. Vascular anatomy should be routinely evaluated in these patients, because variations can impact planning, implementation, and outcomes of invasive interventions on the liver [1–6].

In 1955, Nicholas A. Michels described 10 variations of arterial supply to the liver [7]. This classification became the standard for describing anatomic variants. We examined the variations existing in a West Indian population using Michels' classification. This information is important to optimize the delivery of interventional radiology and hepatobiliary surgical services in persons of West Indian heritage.

## 2. Methods

This study was performed over a 24-month period across two tertiary referral hospitals located in Trinidad and Tobago, an English-speaking island in the West Indies with a population of approximately 1.35 million persons [8]. Both facilities also served as tertiary referral centers for hepatopancreatobiliary (HPB) services to other countries in the West Indies [9]. Therefore, we expected the results of this study to be representative of the wider West Indian population.

At these centers, a multidisciplinary team comprised of radiologists, surgeons, oncologists, gastroenterologists, and pathologists met weekly to review electronic images, discuss therapeutic options, and plan management of patients with HPB diseases. The local institutional review board granted ethical approval to evaluate images for patients discussed at the MDT meetings.

All patients had multiphase computerized tomography (CT) scans using a 64-slice multirow detector CT scanner. A nonionic contrast medium, Ultravist 300® (iopromide), in a volume of 100 mls was routinely administered in all contrast CT abdomen studies using a pressure injector with bolus tracking. In order to reproduce CT acquisition, the technical parameter for image acquisition is outlined in Table 1. Two independent investigators prospectively examined all CT images encountered between August 30, 2016, and September 1, 2018.

The following inclusion criteria were used: all abdominopelvic CT scans with an arterial phase covering the coeliac trunk and superior mesenteric arterial territory; CT angiograms of the abdominal aorta; and all CT scans of the chest that adequately cover the abdomen in the arterial phase. The exclusion criteria were as follows: any CT scans with incomplete demographic data; duplicated scans; scans without adequate arterial phases; scans with poor visualization of arterial anatomy; scans in patients with prior vascular surgery and those with interventional radiology procedures in the upper abdomen.

The arterial variations encountered on CT images were classified according to the system proposed by Michels [7] as outlined in Table 1. Data were recorded in a Microsoft Excel sheet. Descriptive analyses were performed using the SPSS statistical software. Fisher's exact test and two proportion *Z*-tests were used to compare the proportions for each variant with the proportions reported by Michels. A *P*-value <0.05 was considered significant.

## 3. Results

**Figure 1:**
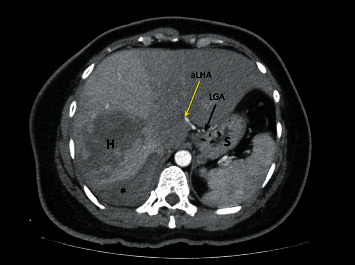
Axial CT images of a patient with a ruptured hemangioma (H) and a subcapsular hematoma (asterix). The left gastric artery (LGA) can be seen medial to the stomach (S). The accessory left hepatic artery (aLHA) originates from the LGA and courses directly into the left liver (type 6 variant).

**Figure 2:**
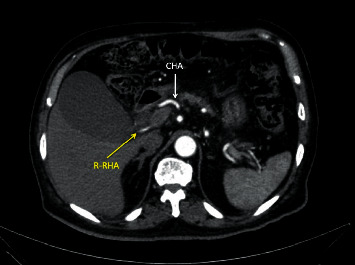
Axial CT images of a patient with a type 3 variant. The normal common hepatic artery (CHA) can be seen coursing laterally in a normal route (white arrow). The replaced right hepatic artery (R-RHA) can be seen coursing toward the right liver posteriorly in the hepatoduodenal ligament, behind the portal vein.

A total of 451 CT scans were examined, and 205 met the inclusion criteria in 205 patients. There were 112 persons (54.6%) with conventional type 1 anatomy. The most common anatomic variants were Michels' type 2 (Figure 1) and Michels' type 3 (Figure 2) patterns. Table 1 outlines the anatomic patterns according to Michel's classification system.

**Figure 3:**
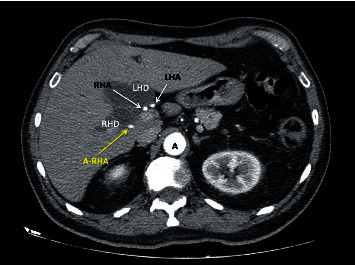
CT images of a patient with a periampullary tumor and a dilated biliary tree. The dilated left (LHD) and right hepatic ducts (RHD) are seen at the liver hilum. Corresponding to this, the proper hepatic artery has divided normally into a left hepatic artery (LHA) and right hepatic artery (RHA) at the hilum. This patient also has an accessory right hepatic artery (A-RHA) as a type 6 variant.

We compared the frequency of the anatomic variants in the West Indian population with those reported in Michels' original report (Table 2). In our population, there was a significantly greater incidence (18.1% vs 11%; *P* 0.044) of type 3 replaced right hepatic arteries (Figure 2) and a significantly lower incidence (2.4% vs 7%; *P* 0.03) of type 6 accessory right hepatic arteries (Figure 3). In the remainder of cases, the distribution of variants was similar to that described by Michels.

## 4. Discussion

In the conventional anatomic descriptions, the common hepatic artery originates from the coeliac trunk and terminates by bifurcating into the proper hepatic and gastroduodenal arteries. The proper hepatic artery then divides into the left and right hepatic arteries at the liver hilum [7]. This classic pattern is reported to be present in 50–60% of persons in international literature [3, 7, 10–12]. The incidence of classic arterial patterns in our population (54.6%) was statistically similar to that reported in the literature.

Type 3 variance (Figure 1), where a replaced right hepatic artery originates from the superior mesenteric artery, was present in 18.1% of West Indians as a sole variance. This is significantly greater than the 11% incidence seen in the Caucasian population studied by Michels [7]. It is also greater than the 12–15% incidence of Michels' type 3 patterns published in more recent image-based studies [10, 12]. We also encountered a significantly lower incidence of type 6 variance (Figure 2), where an accessory right hepatic artery exists. This was seen in 2.4% of West Indians compared to 7% incidence in the Caucasian population studied by Michels [7].

The technical challenges and opportunities presented by a replaced versus an accessory right hepatic artery are quite different both in the context of transplant and resection hepatobiliary surgery. An accessory RHA arising from the SMA may safely be sacrificed during a pancreatoduodenectomy to achieve cancer clearance, whereas a replaced RHA would need to be reconstructed [13]. During a whole organ liver transplant, either of these would need a back-bench preparation; however, as a rule, a replaced RHA is of a larger caliber than an accessory RHA, and thereby, technically easier to reconstruct [13]. The presence of a replaced as opposed to accessory RHA is extremely beneficial in the context of a split liver or a right lobe living donor transplantation for the extra length it provides both during donor liver dissection and recipient implantation [14]. Finally, a replaced RHA has potential benefits for hilar cholangiocarcinoma surgery due to its anatomical course away from the hepatic hilum [13]. Thus, the higher incidence of replaced RHA in the Caribbean population may therefore provide distinct advantages and challenges during hepatobiliary and transplant surgery.

Nevertheless, preoperative mapping of these anomalies is imperative, as technical challenges can be anticipated in advance, and surgical techniques are modified accordingly. Inadvertent or unrecognized injury to these vessels during pancreaticoduodenectomy, major hepatectomies, and liver transplants may lead to significant complications, such as intraoperative hemorrhage, hepatic ischemia, biliary strictures, biliary sepsis, or graft failure.

Although there are different patterns in our population, we are aware that Michels' report [7] had a predominantly Caucasian population. In contrast, Caucasians accounted for only 5% of the population of Trinidad and Tobago [8]. Considering that the population in Trinidad and Tobago is comprised of an equal distribution of persons of East Indian (40%) and Afro Caribbean (40%) descent [8], we compared our data with reports from populations on the African [14–17] and Indian continents [18, 19] to determine whether variations were similar to these populations.

When we performed a literature search for reports that studied variations in populations from the African Continent, we found few case reports detailing replaced right hepatic arteries [17], but only three population-based studies [15, 16, 20] were encountered. Unfortunately, none of the authors used a standardized classification, such as Michels' classification, to report the variations they encountered. However, based on their detailed descriptions, we were able to retrospectively fit some of the patterns into Michels' classification for comparison (Table 3).

The data reported from East and Central Africa [20] was vague, and so, only type 9 variations could be compared, but there was no difference between the populations. Slightly more data was available in Ethiopians [16]. In that population, we noted a greater incidence of type 5 (12.7% vs 4.4%) and type 6 variations (9.09% vs 2.4%) compared to that of West Indians. There was complete data reported from Kenya [15]. We noted that Kenyans had a lower incidence of type 2 variants (1.96% vs 14.6%) but had a higher incidence of type 6 variants (10.8% vs 2.4%) when compared to West Indians. The most consistent finding was a greater prevalence of type 6 variants in the African populations than in the West Indian population.

Generally, the data reported in Indian studies were more robust. A literature search returned two reports that studied variations in populations from South India [18] and North India [19] (Table 4). Thangarajah et al. [18] used CT angiograms to evaluate hepatic arterial anatomy in 200 persons in South India. We found no difference in the incidence of hepatic artery variations between our population and the South Indian population. However, when variations were present, our population had significantly more type 2 (14.6% vs 6%; *P* 0.0044) and type 3 (18.1% vs 8%; *P* 0.0027) variants compared to the South Indians [20]. Sehgal et al. [19] evaluated CT angiograms in 50 persons from Luknow in North India. Similarly, there was no difference in the incidence of hepatic artery variations between our population and the north Indian population. But when variations were present, the West Indians had significantly more type 2 (14.6% vs 4%), but less type 6 variants (2.4% vs 14%).

Overall, the most consistent findings were that the West Indians had a significantly lower incidence of type 6 variants than Indian [19], African [15, 16], and Caucasian populations [7]. The incidence of type 2 variations was also significantly higher in our population than it was in Indian [18, 19] and African [15] populations.

## 5. Study Limitations

The authors considered whether the increased number of replaced right or replaced left hepatic arteries and the corresponding reduced number of accessory left or accessory right hepatic arteries could be a reflection that they were misinterpreting the scans and missing small conventional right or left hepatic arteries. However, all scans were performed on high specification multislice CT scanners with conventional arterial phase protocols and were independently reviewed by two senior radiologists with specialist interests in vascular anatomy. Therefore, we believe that these are true findings.

It was also considered whether it might be possible to identify whether the high incidence of variants might be present within patients from a particular ethnic group. However, due to the nature of this study, it was not possible to make this distinction. This should be considered in future studies on this population.

## 6. Conclusion

Although 54.6% of persons in the West Indies have conventional vascular anatomy, the distribution of arterial variants is quite different to that seen in other regions. Healthcare professionals performing hepatobiliary interventions in the West Indies must be aware of these differences in order to minimize morbidity during their interventions.

## Figures and Tables

**Table 1:** Technical parameters for acquisition of CT images.

Parameter	Details
CT scanner detail	Images were acquired on one of the following CT scanners, all utilizing multidetector technology:
	(i) Siemens somatom definition 64-slice scanner
	(ii) Hitachi scenaria view 128-slice scanner
	(iii) General electric lightspeed VCT 64-slice scanner
Image acquisition	Isotropic imaging was utilized in all acquisitions, with 0.625 mm slices acquired, with images reviewed utilizing multiplanar reformats with a reconstructed slice thickness of 1.25 mm
Contrast tracking	Images utilized in this study were acquired in the arterial phase of contrast enhancement, utilizing bolus tracking software, with the region of interest centered over the lower thoracic (descending) aorta
Contrast phase	A single arterial phase acquisition was performed, followed by portal venous phase images

**Table 2:** The Anatomic Distribution of Hepatic Arteries in an Unselected West Indian Population compared to Michels' original report.

Type	Michels' description (1995)	Eastern Caribbean *n* (%)	Michels [7] *n* (%)	*P* value
1	Normal: Common hepatic artery from the coeliac trunk, branching into the proper hepatic artery (with terminal branching to the right and left hepatic artery) and the gastroduodenal artery	112 (54.6%)	110 (55%)	0.9410
2	Replaced left hepatic artery from the left gastric artery	30 (14.6%)	20 (10%)	0.1564
3	Replaced right hepatic artery from the superior mesenteric artery	37 (18.1%)	22 (11%)	0.0444
4	Replaced right hepatic artery and left hepatic artery	2 (0.98%)	2 (1%)	0.9802
5	Accessory left hepatic artery	9 (4.40%)	16 (8%)	0.1313
6	Accessory right hepatic artery	5 (2.40%)	14 (7%)	0.0300
7	Accessory right and left hepatic arteries	2 (0.98%)	2 (1%)	0.9802
8	Replaced right hepatic artery and accessory left hepatic artery OR replaced left hepatic artery and accessory right hepatic artery	3 (1.50%)	4 (2%)	0.6787
9	Common hepatic artery from the superior mesenteric artery	5 (2.40%)	9 (4.5%)	0.2563
10	Common hepatic artery from the left gastric artery	0 (0.00%)	1 (0.5%)	0.3107
Total		205	200	

Data were taken from Michels NA. Blood supply and anatomy of the upper abdominal organs with a descriptive atlas. Philadelphia: Lippincott, 1955.

**Table 3:** A comparison of hepatic artery variations in the West Indies and Africa.

Type	Michels' description	West Indies	East-Central Africa ^*∗*^ [20]	*P*	Kenya ^*∗∗*^[15]	*P*	Ethiopia ^*∗∗∗*^[16]	
1	Normal anatomy	112 (54.6%)	N/A	—	74 (72.5%)	0.0025	N/A	—
2	Replaced left hepatic artery from the left gastric artery	30 (14.6%)	N/A	—	2 (1.96%)	0.0006	12 (10.9%)	0.3538
3	Replaced right hepatic artery from the superior mesenteric artery	37 (18.1%)	N/A	—	0 (0.00%)	—	9 (8.18%)	0.0181
4	Replaced right hepatic artery and left hepatic artery	2 (0.98%)	N/A	—	0 (0.00%)	—	N/A	—
5	Accessory left hepatic artery	9 (4.40%)	N/A	—	10 (9.80%)	0.0637	14 (12.7%)	0.0067
6	Accessory right hepatic artery	5 (2.40%)	N/A	—	11 (10.8%)	0.0019	10 (9.09%)	0.0082
7	Accessory right and left hepatic arteries	2 (0.98%)	N/A	—	0 (0.00%)	—	0 (0.00%)	—
8	Replaced right hepatic artery and accessory left hepatic artery OR replaced left hepatic artery and accessory right hepatic artery	3 (1.50%)	N/A	—	0 (0.00%)	—	N/A	—
9	Common hepatic artery from the superior mesenteric artery	5 (2.40%)	8 (5%)	0.2236	5 (4.90%)	0.2522	0 (0.00%)	—
10	Common hepatic artery from the left gastric artery	0 (0.00%)	N/A	—	0 (0.00%)	—	0 (0.00%)	—
	Not classified OR does not fit Michels' description	0	160	—	0 (0.00%)	—	N/A	—
Total		205	168	—	102		110	

N/*A* = data not available and/or poorly described, not allowing interpretation using Michels' Classification. Data were taken from ^*∗*^Ibingira CBR. Gross Anatomical Variations And Congenital Anomalies Of Surgical Importance In Hepatobiliary Surgery In Uganda. East And Central African Journal Of Surgery. 2006; 12 (1): 93–98. ^*∗∗*^Tharao MK, Saidi H, Kitunguu P, Ogengo AJ. Variant Anatomy of the Hepatic Artery in Kenyans. Eur *J* Anat. 2007; 11 (3): 155–161. ^*∗∗∗*^Futara *G*, Ali A, Kinfu Y. Variations of the hepatic and cystic arteries among Ethiopians. Ethiop *J* Med. 2001; 39 (2):133–142.

**Table 4:** A comparison of hepatic artery variations in the West Indies and India.

Type		West Indies	Thangarajah et al. [18] (South India) ^*∗*^	*P*	Sehgal et al. [19] (North India) ^*∗∗*^	*P*
1	Normal anatomy	112 (54.6%)	114 (57%)	0.6317	31 (62%)	0.3467
2	Replaced left hepatic artery from the left gastric artery	30 (14.6%)	12 (6%)	0.0044	2 (4%)	0.0418
3	Replaced right hepatic artery from the superior mesenteric artery	37 (18.1%)	16 (8%)	0.0027	5 (10%)	0.1689
4	Replaced right hepatic artery and left hepatic artery	2 (0.98%)	2 (1%)	0.9802	1 (2%)	0.5470
5	Accessory left hepatic artery	9 (4.40%)	17 (8.5%)	0.0916	2 (4%)	0.9031
6	Accessory right hepatic artery	5 (2.40%)	2 (1%)	0.2666	7 (14%)	0.0005
7	Accessory right and left hepatic arteries	2 (0.98%)	2 (1%)	0.9802	0 (0)	0.4832
8	Replaced right hepatic artery and accessory left hepatic artery OR replaced left hepatic artery and accessory right hepatic artery	3 (1.50%)	3 (1.5%)	0.9757	0 (0)	0.3895
9	Common hepatic artery from the superior mesenteric artery	5 (2.40%)	3 (1.5%)	0.4972	0 (0%)	0.2647
10	Common hepatic artery from the left gastric artery	0 (0.00%)	1 (0.5%)	0.3107	0 (0%)	—
	Not classified OR does not fit Michels' description	0	28		2 (4%) replaced RHA from CA	—
Total		205	200		50	

Data were taken from ^*∗*^Thangarajah A, Parthasarathy R. Celiac *Axis*, Common Hepatic and Hepatic Artery Variants as Evidenced on MDCT Angiography in South Indian Populations. *J* Clin Diagn Res. 2016; 10 (1):1–5. ^*∗∗*^Sehgal *G*, Srivastava AK, Sharma PK, Kuman N, Singh R. Variations of Extra-Hepatic Segments of Hepatic Arteries: A Multislice Computed Angiography Study. Int *J* Scientific Res Publications. 2013; 3 (2): 1–8.

## Data Availability

The data are available from the corresponding author upon request.
